# Practising pastoralism in an agricultural environment: An isotopic analysis of the impact of the Hunnic incursions on Pannonian populations

**DOI:** 10.1371/journal.pone.0173079

**Published:** 2017-03-22

**Authors:** Susanne E. Hakenbeck, Jane Evans, Hazel Chapman, Erzsébet Fóthi

**Affiliations:** 1 Department of Archaeology and Anthropology, University of Cambridge, Cambridge, United Kingdom; 2 NERC Isotope Geosciences Laboratory, Keyworth, Nottingham, United Kingdom; 3 Department of Earth Sciences, University of Cambridge, Cambridge, United Kingdom; 4 Hungarian Natural History Museum, Budapest, Hungary; University of Florence, ITALY

## Abstract

We conducted a multi-isotope study of five fifth-century AD cemeteries in modern-day Hungary to determine relationships between nomadic-pastoralist incomers—the historically documented Huns and other nomadic groups—and the sedentary agricultural population of the late Roman province of Pannonia. Contemporary historical sources describe this relationship as adversarial and destructive for the late Roman population, but archaeological evidence indicates high levels of hybridity between different groups. We undertook carbon, nitrogen, strontium and oxygen isotope analyses of bone collagen, dentine and tooth enamel at Keszthely-Fenékpuszta, Hács-Béndekpuszta, Győr-Széchenyi Square, Mözs and Szolnok-Szanda to examine these relationships through past subsistence practices. The patterns at all sites indicate medium to high animal protein consumption with little evidence for a significant contribution of aquatic resources. All populations relied to a great extent on C4 plants, most likely millet. Within each population, diet was heterogeneous, with significant variations in terms of animal protein and C3 and C4 plant consumption. High levels of intra-population and individual variability suggest that populations made use of a range of subsistence strategies, with many individuals exhibiting significant changes over their lifetimes. Rather than being characterised only by violence, the historically-documented influx of nomadic populations appears to have led to widespread changes in subsistence strategies of populations in the Carpathian basin. Nomadic-pastoralist groups may have switched to smaller herds and more farming, and, conversely, local populations may have integrated with a new economic system based on animal herding.

## Introduction

The fifth century AD was a period of far-reaching changes along the northern and eastern frontier of the Roman empire. Historical sources tell of warbands of nomads on horses, which they call Huns or Scythians, attacking settlements all along the Danube frontier and occasionally extending as far as northern Italy or France. These incursions have been interpreted as the initial destabilisation that set in motion the breakdown of the western Roman empire [1]. The narrative surrounding these events frequently emphasises the fundamental cultural difference between these nomads and the settled populations of the late Roman provinces.

Archaeologically, however, these dichotomies are less obvious. Few sites provide evidence of violence and it is difficult to link these destruction layers to historically recorded Hunnic attacks [2, 3]. Conversely, much of the material culture evidence from burials show high levels of hybridity—mixing of late Roman and non-Roman practices—sometimes in the same cemetery. Different ethnic groups cannot clearly be distinguished. Certain types of material culture—bronze cauldrons, bronze mirrors, diadems, remains of composite bows—and practices such as skull modification have been associated with Huns or other nomadic-pastoralist groups because of their typological association with the north-Pontic areas (north of the Black Sea) and central Asia [4, 5]. Yet, while this material occurs in eastern and central Europe, it rarely forms an assemblage so cannot be linked unequivocally to these mobile groups.

Isotope analysis allows us to access directly the behaviours of individuals, both in terms of their mobility and of their diet. Here we aim to investigate the evidence for mobility—pointing to a nomadic lifestyle—using ^86^Sr/^87^Sr and δ^18^O ratios in tooth enamel, combined with evidence for dietary variability provided by δ^15^N and δ^13^C ratios in teeth and bone—possibly distinguishing pastoral and agricultural diets. The analysis of multiple samples of different tissue types from the same individual has been shown to provide greater resolution to dietary variability, because it allows us to track lifetime changes in the circumstances of individuals [6, 7]. Such a biographical approach may reveal whether people altered their subsistence practices in changing social and environmental contexts or whether they consistently followed particular models of subsistence. Put simply, we can explore whether pastoralists could become famers, and, by extension, whether ‘Huns’ could become settled dwellers of Pannonia.

In undertaking this study we aim to shed new light on the complex and dynamic relationships of populations across the late Roman frontier zone, from within the Roman province of Pannonia to sites in the Great Hungarian plain to determine the impact of the Hunnic incursions on this part of the world.

## Determining the isotopic signatures of pastoral and agricultural diets

The diets of agricultural populations of early medieval western and central Europe are characterised by great homogeneity. Populations consumed a terrestrial diet with a medium to high amount of animal protein and limited input of aquatic or marine resources, and they relied largely on C3 plants [8–12].

The diet of Eurasian mobile pastoralists of the same period has been less thoroughly investigated, with the majority of studies focusing on the Bronze and Iron Ages [13–17]. Archaeobotanical and isotopic studies show a transition towards increasing millet cultivation from the second millennium onwards. This was identified as early as 5900 BC in northern China, by about 1800 BC in Kazakhstan and about 1500 BC in southern Siberia [13, 17, 18]. Millet then remained an important grain for central Asian populations throughout the Iron Age and into the first millennium AD. It is drought-tolerant, has a high yield per plant and has a short growing season (c. six weeks), making it a useful crop for mobile populations either as food or fodder [18]. Central and Inner Asian populations were frequently more enriched in δ^15^N compared to European populations. This may have been due to increased consumption of animal protein, as would be expected of pastoralist communities, but fish has also been shown to play a significant role [19]. However, comparisons of humans and local fauna have also shown that the aridity of the environment may have resulted in cumulative enrichment of the ecosystem [13, 14, 16, 20, 21].

## Methods

Carbon and nitrogen isotope ratios in bone collagen and dentine, reported as δ^13^C and δ^15^N, allow us to study the relative contribution of terrestrial, marine and freshwater resources to diet. Carbon isotope ratios distinguish dietary contributions from C3 and C4 plants, since these plants have isotopic ratios that do not overlap [22, 23]. These values are enriched by approximately 5‰ from diet to body tissue. Most plants utilise the C3 pathway, while the C4 pathway is used by plants in hot and dry climates, mostly grasses and sedges. The key cultivars in pre-colonial Europe are millet, amaranth and sago.

A comparison of δ^13^C values in enamel apatite and dentine can reveal information about relative contributions of ^13^C from protein sources or from the diet as a whole, since the carbon in dentine collagen is preferentially routed from protein in the diet, whereas carbon in the carbonate component of apatite reflects blood-dissolved inorganic carbon derived mainly from carbohydrates and lipids [24]. If the source and amount of dietary protein is roughly the same within a population, there is a strong correlation between δ^13^C_collagen_ and δ^13^C_apatite_ [25–27]. However, where the protein source is varied, for example by including marine foods or animals foddered on C4 plants, this relationship is no longer straightforward [28]. A comparison of δ^13^C_collagen_ and δ^13^C_apatite_ can reveal the degree to which δ^13^C ratios reflect direct routing from plants or through animal protein. Unlike collagen, which only reflects the protein contribution to diet, δ^13^C_apatite_ is sensitive to C4 contributions from the whole diet that may be obscured in collagen.

Nitrogen isotope values allow us to determine the relative amount of animal protein (meat or milk) consumed by an organism. δ^15^N is enriched by about 3‰ with each trophic level [29, 30], but the enrichment can be as high as 6‰ in some instances [31]. Both freshwater and marine organisms are enriched in ^15^N, though freshwater fish in particular are strongly affected by their ecological context [32].

Strontium and oxygen isotope ratios are used to determine whether an individual grew up where he or she was buried [33]. ^87^Sr/^86^Sr values in organisms reflect those of the underlying geology [34]. Bioavailable strontium enters the food chain through water and is incorporated in bone and enamel apatite [35, 36]. Due to its regular crystal structure, which limits the ion exchange after tooth formation, enamel apatite is not subject to diagenetic change in the burial environment [37]. Isotopic signatures in teeth thus provide an indication of childhood residence and can be contrasted with local bioavailable ^87^Sr/^86^Sr values to determine if there was a change in residence since childhood [33]. However, only individuals who grew up in a location with different geological strontium isotope ratios can be identified as non-local. Strontium analysis can therefore only indicate a non-local upbringing, but it cannot definitely indicate a local upbringing.

Oxygen isotope ratios (δ^18^O) also vary geographically, primarily due to differences in temperature, becoming more depleted from the equator to the poles and with increasing altitude [38]. Across Eurasia values are also depleted from west to east, along with the prevailing winds. Via an offset, organisms reflect the isotopic value of drinking water, which in turn usually reflects the values of rainwater. Attempts have been made to develop a conversion algorithm for the offset from drinking water to body tissue for different species, but these have not been fully satisfactory [39, 40]. In many published studies, intra-population variation of oxygen isotope ratios is greater than large-scale geographical variations of precipitation (e.g. from Scandinavia to the Mediterranean) [41]. This may be due to water sources other than rainwater and changes in isotopic ratios due to heating water [42]. In this study we therefore restrict ourselves conservatively to inter-population comparisons to examine variability and identify outliers. To enable comparison with other published data, we converted our data, which was measured δ^18^O relative to VPDB in enamel carbonate, to phosphate values relative to VSMOW, using conversion equations provided in [43, 44].

The full analytical methods are described in the supporting information (S1 Text).

### Lifetime changes

Due to differential remodelling of body tissues, different tissue types can provide ‘windows’ into specific periods in a person’s life. Human teeth erupt following a regular pattern [45], with roots (dentine) forming slightly later than the crowns. Neither enamel nor primary and secondary dentine is remodelled, so isotopic values from teeth reflect the period when they were formed. Incremental samples of tooth roots have been shown to provide near annual resolution, as tooth roots form at about 1 mm per year [46, 47]. We chose second molars (M2) or second pre-molars (P2) to represent childhood and early juvenility and third molars (M3) to represent juvenility and early adulthood (S1 Table).

Contrasting with teeth, bone collagen is constantly remodelled [48]. Ribs in particular are subject to considerable muscular stresses and therefore have a shorter turnover period than other bones. Isotopic values from ribs thus represent a period towards the end of a person’s life, commonly assumed to be less than ten years, though contributions from earlier years are possible [49].

Taking multiple samples from the same individuals opens up the unique possibility of examining changes in diet and residence of individuals over the course of their life [6, 7]. This is of particular interest when studying nomadic-pastoralists in an agricultural environment, where a change in residence may coincide with a change in diet, because it can indicate to what extent individuals adapted to new social and environmental contexts.

## Sites

**Fig 1:**
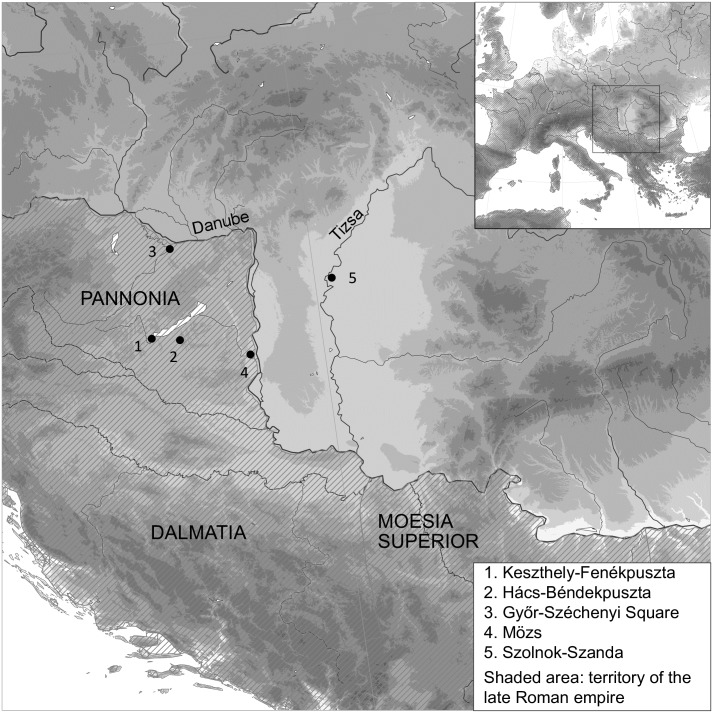
Map showing the location of the sites on either side of the late Roman frontier. Roman provinces map data adapted from the Ancient World Mapping Centre (Creative Commons Attribution-NonCommercial 3.0 Unported); coast line and river data from the GSHHG [50]; elevation data from the GMTED2010 [51].

Five sites were chosen to cover a large area across the frontier zone, from the heartland of the province of Pannonia to the Great Hungarian Plain beyond the river Tisza (Fig 1). Grave goods date them to the fifth century AD and they all contain some evidence for autochthonous as well as non-local practices (e.g. grave goods, grave construction or skull modification). Detailed information about the samples and the sampling strategy is presented in the supporting information (S2 Text).

The Roman fort of Keszthely-Fenékpuszta (46.707196 N, 17.246788 E) is situated on the western shore of Lake Balaton, well away from the Danube frontier. It was founded in the mid fourth century AD and continued to be in use in some form into the ninth century AD. It is considered part of a group of internal fortifications of Pannonia [52–54]. To the south of the walls there are extensive groups of cemeteries, dating from the entire period the site was in use [55, 56]. About 250 m farther to the south-east there is a distinctive group of thirty-one inhumations dating to the fifth century AD, excavated from 1976 to 1980 [57]. The grave goods have been associated with the region around the Dnepr and Crimea, as well as exhibiting the heterogeneity common to mid fifth-century cemeteries in Pannonia [57, 58]. Twenty-eight individuals had modified skulls [59–61]. This group was therefore sampled for our study.

Compared with the rather more cosmopolitan nature of Keszthely-Fenékpuszta, the nearby cemetery of Hács-Béndekpuszta (46.660274 N, 17.713328 E) was chosen because it reflected a small group, possibly a family. Hács-Béndekpuszta is not far from Keszthely-Fenékpuszta, c. 15 km south of Lake Balaton [62]. It was discovered in 1934 and excavated in the 1950s. This revealed twenty-nine graves, though the full extent of the cemetery is unknown, since building work in the 1940s caused some losses. Grave goods date this cemetery to the last third of the fifth century AD [63]. Grave furnishings are representative of the region, including some items that have typological links with areas as far away as southern Germany. One individual (grave 23) had a modified skull [64]. Hács-Béndekpuszta is also notable because one of the disturbed graves (grave 5) contained rare fragments of texts from the Gothic bible, written on lead sheets that were possibly used as an amulet [65]. This is important evidence of Gothic missionary activity in middle Danube region.

The cemeteries of Győr-Széchenyi Square (47.688145 N, 17.634333 E) and Mözs are located on the banks of the Danube and thus directly on the frontier. The modern town of Győr is situated at the confluence of the river Rába and the Moson Danube (Little Danube). A number of excavation campaigns in Széchenyi Square, in the city centre, revealed the remains of the Roman vicus of Arrabona [66, 67]. Following its abandonment, a cemetery was established there [68, 69]. Seventy-nine graves were dug into a thick layer of dark earth or possibly river silt, suggesting that the vicus had been abandoned some time earlier [69]. Some of the graves were brick-lined, while four, or possibly five, individuals had modified skulls. The grave furnishings overall were fairly limited and exhibit both late Roman and ‘foreign’ traits [67, 69]. Based on grave good typologies, the cemetery was in use during the second and third quarters of the fifth century AD and possibly later [63].

The cemetery at Mözs (46.401353N, 18.716669 E) lies on a low rise on the banks of a now abandoned channel of the Danube. In 1961 twenty-eight graves were excavated [70]. These are the subject of the present study. In the 1990s a further sixty-eight graves were discovered south of the original excavation in advance of the construction of the M9 motorway [71]. The cemetery was fully excavated, as well as its associated settlement. Burial practices are very heterogeneous: some graves are lined with bricks, a late Roman practice, while others had built-in niches, a practice that is associated with non-Roman groups. Like Győr, the cemetery was in use during the second and third quarters of the fifth century [63]. The grave goods exhibit a wide range of typological influences. In the northern group nine individuals had modified skulls, and more than forty in the cemetery as a whole.

The cemetery of Szolnok-Szanda (47.140898 N, 20.194801 E), finally, lies far beyond the Roman frontier on the banks of the river Tizsa in the Great Hungarian Plain. It was discovered in 1952 and excavated more systematically from 1955 to 1957 [72]. This cemetery is part of a cluster of cemeteries in the region that are associated with Gepids who were a group known from written sources to have been allied to the Huns [73]. However, no material culture is uniquely specific to this region and can therefore clearly be identified as Gepid [74]. Instead, grave construction and furnishings are part of a regionally widely shared practice. Six individuals of 206 graves had modified skulls. Based on grave good evidence, the cemetery was in use in the fifth and sixth centuries.

## Geological and environmental background

The Pannonian basin is largely made up of deep loess deposits. They began to be formed during MIS 27–17 (1 Ma-700 ka), with the last big loess deposition event taking place during the Last Glacial Maximum and the Younger Dryas [75]. Secondary loess deposits accumulated through alluvial transportation. This means that much of the interior Pannonian basin is geologically very homogeneous, though there are some exceptions.

The site of Keszthely-Fenékpuszta is located at the western end of Lake Balaton on Holocene paludial sediments. A hilly area about 10 km to the north is formed by the Somló Formation, made up of sand, siltstone and clay beds, and dating from the Upper Miocene (11–5 Ma). The Keszthely Mountains, part of the Bakony mountains north of the south-western end of the lake and closest to Keszthely-Fenékpuszta, are characterised by Upper Triassic (230–200 Ma) marine dolomite and limestone [76]. The area around Hács-Béndekpuszta is dominated by Pleistocene loesses [76]. The Győr region is characterised by Holocene paludial sediments and fluvial sand and gravels [76]. Similarly, Mözs is situated between Pleistocene loesses and the Holocene Danubian floodplain [76]. About 10 to 15 km to the southwest the floodplain rises to the Szekszárd Hills. They are of Mórágy granite, dating to 320 to 310 Ma [76], overlain by deposits of the Somló Formation to the north. Szolnok, finally, is located in the Tisza floodplain which is made up of Holocene fluvial deposits.

Within the Roman province of Panonnia, there is extensive evidence of farming and animal husbandry. A study of pollen evidence in south-west Hungary indicates increasing deforestation since the Iron Age resulting in the formation of open meadows [77]. During the Roman period grasslands were used for animal husbandry, but there is also an increase in cereal cultivation. In Keszthely-Fenékpuszta the main cereal crops were six-rowed barley, bread wheat and also rye, while lentils, peas and beans also played an important role [78]. The area around the lake was used to cultivate cereals (predominantly barley and wheat), legumes and also grapes [79]. Keszthely-Fenékpuszta has an abundance of fauna from all periods of the site [80]. The majority are local domesticates (predominantly pig and cattle) and wild fauna such as wild boar, aurochs and red and roe deer, but there is also a contribution of imported species, notably the phalanx of a Bactrian camel [80].

The environment in the Great Hungarian Plain is characterised by a mosaic of different types of steppe habitats that have also been subject to considerable changes over the course of the Holocene [81]. From the Neolithic onwards land was increasingly used for agriculture, leading to extensive anthropogenic landscape changes [82–84]. Archaeobotanical evidence from the third- to fourth-century settlement site of Kiskundorozsma-Nagyszék in Csongrád County suggests a highly diverse environment consisting of waterlogged meadows, pastures and open woodland [78]. Indeed, extensive areas of the floodplains of the river Tizsa were permanently or periodically inundated [78]. Prior to the eighteenth century, land-use typically switched between crop cultivation and grazing in multi-year cycles [81]. Millet was cultivated here in greater amounts than within Pannonia and continued to be an important crop in post-Roman periods [85].

## Results

### Sample preservation

The full results of human and faunal samples, together with relevant osteological information, are listed in S2 Table. Samples generally produced collagen of good quality. The atomic C/N ratios were mostly between 3.1 and 3.4, well within the range of 2.9 to 3.6 considered to be indicative of good collagen preservation [86]. Samples that produced C/N ratios outside of 2.9 to 3.6 were excluded. These were mostly fish bones and other fauna. Collagen and dentine samples yielded carbon between 30 and 50% and nitrogen between 11 and 18%, falling in the range of modern human collagen (40–50% carbon and 15–18% nitrogen), as defined by Ambrose [87]. All samples yielded sufficient ppm of strontium.

### Fauna

Cattle and ovicaprids were used to establish a dietary baseline for the human samples, since only they were present at all sites and were likely a dietary mainstay for both mobile and settled societies (S2 Fig). Summary data are given in S3 Table. A one-way ANOVA (*F*_*δ13C*_ (3, 42) = 1.651, *p* = 0.192; *F*_*δ15N*_ (3, 42) = 2.631, *p* = 0.062) shows that values for cattle and ovicaprids at the four sites with animal bones are not significantly different. Any variability in the human results is therefore due to human dietary choices rather than regional ecological variation. Scatterplots of all fauna and human samples at each site are presented in the supplementary section (S3 Fig).

Fishbones were sourced from Lake Balaton, as well as the rivers Rába, Danube and Tisza. Isotopic values of the same species differ widely at different locations. Lacustrine fish species have been shown to have variable isotopic values due to the variability of the freshwater ecosystems in which they live [32]. Catfish are particularly variable in both δ ^15^N and δ ^13^C values. The isotopic values of the different ecosystems within the Danube are as yet insufficiently characterised, but this variability may be due to different isotopic compositions of the riverine carbon source as well as the importance of aquatic CAM plants in the foodweb [88].

### Comparison of diet at different sites

**Fig 2:**
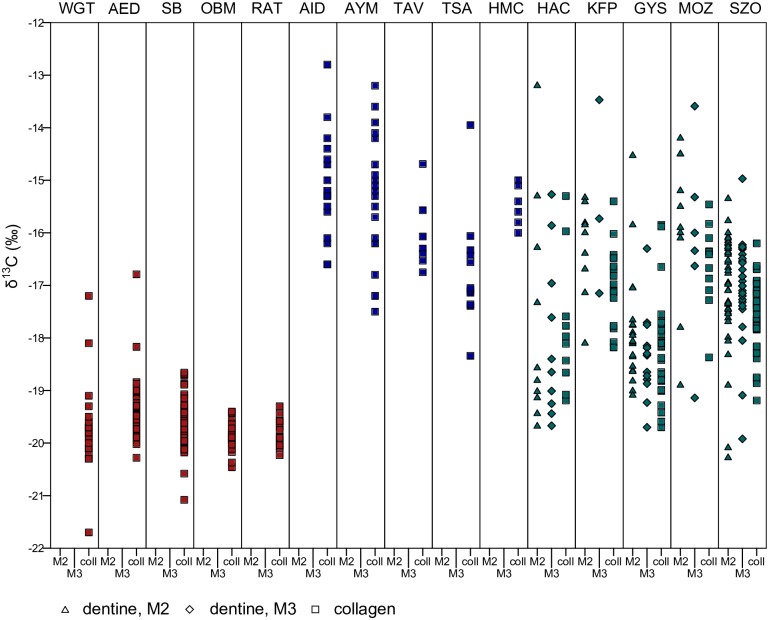
Comparison of δ^13^C values at different sites shows the Hungarian sites falling between German and Inner Asian ones. The colours indicate the general location of the sites. Red: Germany; blue: Inner Asia; green: Hungary (data from this study). Comparative data taken from published literature. Key to sites: WGT—Weingarten (fifth to sixth century AD, Germany) [9]; AED—Altenerding (fifth to sixth century AD, Germany) [10]; SB—Straubing (fifth to sixth century AD, Germany) [10]; OBM—Obermöllern (fifth to sixth century AD, Germany) [11]; RAT—Rathewitz (fifth to sixth century AD, Germany) [11]; AID—Ai-Dai (fifth to second century BC, western Siberia) [14]; AYM—Aymyrlyg (eighth to fifth century BC, western Siberia) [14]; TAV—Tavan Tolgoi (thirteenth to fourteenth century AD, eastern Mongolia) [89]; TSA—Tsaganchuluut (thirteenth to fourteenth century AD, eastern Mongolia) [89]; HMC—Hets Mountain Cave (fifteenth to sixteenth century AD, eastern Mongolia) [90]; KFP—Keszthely-Fenékpuszta; HAC—Hács-Béndekpuszta; GYS—Győr; MOZ—Mözs; SZO—Szolnok-Szanda. Columns are labelled as coll—collagen, M2 –second molar and M3 –third molar.

**Fig 3:**
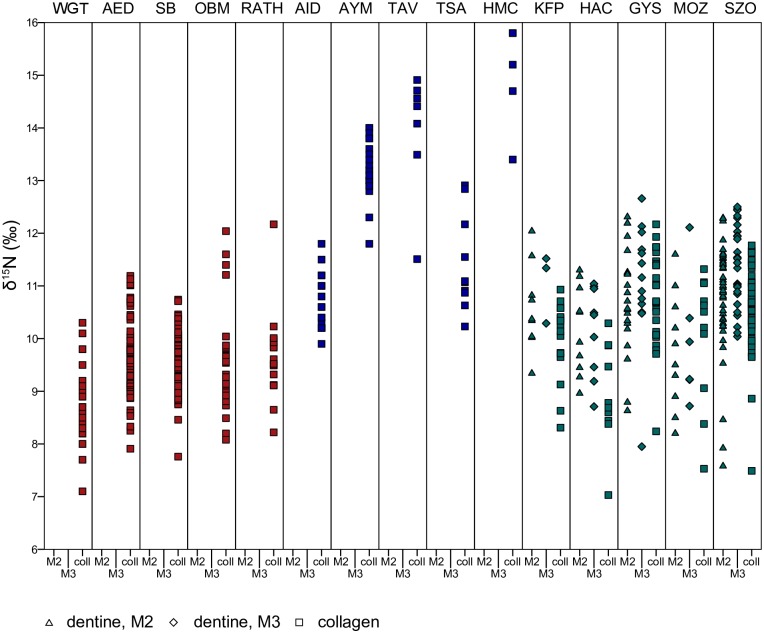
Comparison of δ ^15^N values at different sites shows the Hungarian sites falling between German and Inner Asian ones. The colours indicate the general location of the sites. Red: Germany; blue: Inner Asia; green: Hungary (data from this study). Key to sites: WGT—Weingarten (fifth to sixth century AD, Germany) [9]; AED—Altenerding (fifth to sixth century AD, Germany) [10]; SB—Straubing (fifth to sixth century AD, Germany) [10]; OBM—Obermöllern (fifth to sixth century AD, Germany) [11]; RAT—Rathewitz (fifth to sixth century AD, Germany) [11]; AID—Ai-Dai (fifth to second century BC, western Siberia) [14]; AYM—Aymyrlyg (eighth to fifth century BC, western Siberia) [14]; TAV—Tavan Tolgoi (thirteenth to fourteenth century AD, eastern Mongolia) [89]; TSA—Tsaganchuluut (thirteenth to fourteenth century AD, eastern Mongolia) [89]; HMC—Hets Mountain Cave (fifteenth to sixteenth century AD, eastern Mongolia) [90]; KFP—Keszthely-Fenékpuszta; HAC—Hács-Béndekpuszta; GYS—Győr; MOZ—Mözs; SZO—Szolnok-Szanda. Columns are labelled as coll—collagen, M2 –second molar and M3 –third molar.

Figs 2 and 3 show the δ^13^C and δ^15^N values of each of the Hungarian sites, plotted against five early medieval cemeteries in Germany dating from the fifth and sixth centuries AD and five sites in Siberia and Mongolia dating from the Iron Age and the Mongol Period. The two groups of sites were chosen to represent sedentary agricultural populations on the one hand and pastoral groups on the other, based their associated archaeological and historical context. These populations are used as sedentary-agricultural and nomadic-pastoral end points to determine the extent to which populations in the Pannonian basin pursued either subsistence strategy.

The δ^13^C values of the German sites are tightly clustered, with some outliers. Their δ^15^N values exhibit greater ranges, and here too there are some outliers. These populations can be considered largely sedentary and agricultural, consuming medium amounts of animal protein and mostly C3 plants. Dietary variability is limited. In contrast, the Siberian and Mongolian individuals are enriched in δ^13^C by 4 to 5‰, exhibiting values that indicate considerable though not exclusive reliance on C4 plants. The δ^15^N values are very variable, with Ai-Dai and Tsagaanchuluut falling between about 10 to 13‰, while the populations of Tavan Tolgoi and Hets Mounain Cave range from c. 13.5‰ to almost 16‰. The reasons for this variability are not entirely clear, but likely relate to environmental differences. It has been suggested that enrichment of δ^15^N values could have been caused by the aridity of steppe environments [16, 91] or by localized higher growth season temperatures [89]. We therefore cannot unequivocally relate these very elevated δ^15^N values to greater consumption of animal protein, as might be expected of pastoral populations, though it likely was the cause of some degree of enrichment.

The Hungarian sites are considerably enriched in δ^13^C compared to the German sites though lower than the Inner Asian ones, suggesting a significant, but not exclusive, input of C4 plants. The δ^15^N values are also elevated relative to the German sites, indicating a greater contribution of animal protein to the diet. Levels of aridity and temperature in Hungary and Germany are similar enough that we can attribute this variation to differential protein consumption. The δ^13^C ranges at the Hungarian sites are also greater than at the German sites but comparable to the Inner Asian sites, while the the δ^15^N ranges are greater than both. The distributions are loosely clustered with a number of outliers in both directions. The data ranges are extended with the addition of the dentine data (which was not available for the German and Inner Asian sites). However, even the collagen data include a number of outliers.

The Hungarian sites are therefore characterised by a diet that lies somewhat between the sedentary-agricultural diet of early medieval Germany and the nomadic-pastoralist diet of the Inner Asian sites. Populations consumed a medium to high amount of animal protein and high amounts of C4 plants. The extensive data ranges suggest that some individuals at all sites consumed a more a more agricultural diet, others a more clearly pastoral diet, and many a mixture of both. There is also no clear regional pattern—the four sites within Pannonia are not fundamentally different from Szolnok in the Great Hungarian Plain.

### Lifetime changes in diet

**Fig 4:**
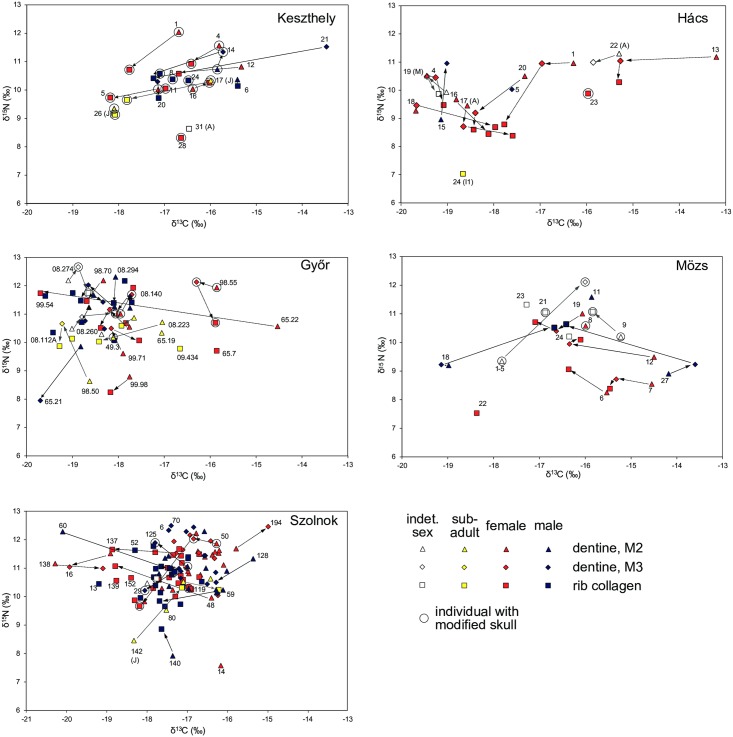
Scatter plots of δ^13^C and δ^15^N values of dentine and bone collagen at the five sites. Changes of the course of the lifetime of individuals are indicated with arrows. Data points are labelled with grave numbers and information about age at death.

Fig 4 shows changes in diet over the lifetime of those individuals where multiple samples were taken in terms of δ^13^C and δ^15^N of dentine and bone collagen. At Keszthely a group of six or seven individuals underwent a significant shift in diet over the course of their lives, changing from greater reliance on C4 plants and animal protein to a diet much reduced in C4 and lower in protein. In Hács individuals also exhibit considerable shifts in diet, though the patterns are less uniform. Dietary changes between tooth dentine and rib collagen mostly manifest as a depletion in δ ^15^N of -0.8 to -2.2‰ (HAC 1, 13, 16, 18, 20). In terms of Δ^13^C offsets between dentine and rib collagen, individuals fall into roughly two groups: some are becoming more enriched—HAC16, 17, 18 by 0.7 to 1.7‰ –while others are more depleted—HAC1, 13, 20 by -1.1to -1.5‰. It is clear that some individuals were subject to quite extreme changes both in their animal protein consumption where a reduction is evident, and in terms of the vegetal component of the diet.

In Győr the picture is more confused. Many individuals exhibit Δ^15^N and Δ^13^C changes between dentine and collagen of up to 0.5‰ in either direction. Quite possibly this is a reflection of normal dietary variation over a person’s lifetime, without being indicative of more widespread social changes. However, a few individuals have significant offsets between childhood and adulthood, most notably GYS65.22. This represents a shift from a large proportion of C4 in the diet to almost none.

In Mözs it is possible to identify two directions of change in δ^13^C values: MOZ6, 7, 12 and 27 are becoming more depleted, while MOZ1-5 and 18, on the other hand, are becoming more enriched. These two small groups had a childhood diet was lower in animal protein. One group consumed significantly higher amounts of C4 plants, MOZ27 in particular being highly enriched in M2 and M3. The other group consumed only a limited amount of C4 plants.

In Szolnok, like in Győr, no clear pattern can be identified. There is a considerable amount of low-level variability of 0.2 to 0.4‰ between different teeth and collagen. In Szolnok the majority of the population consumed high levels of protein and significant amounts of C4 plants. A number of individuals were subject to considerable changes in diet over their lifecourse, but these changes are highly individualized and appear not determined by any group behavior.

At all five sites there is considerable dietary variation over the lifetimes of individuals, with some exhibiting quite extreme shifts over short periods of time. There are no obvious demographic patterns, such as gender or age at death, that could explain such shifts. There is also no relationship between isotopoic variability and specific items of material culture or burial practice, nor are individuals with modified skulls treated differently. However, at Keszthely, Hács and Mözs it may be possible to identify sub-groups that were subject to similar lifetime changes.

### Comparison of protein and whole diet carbon sources

**Fig 5:**
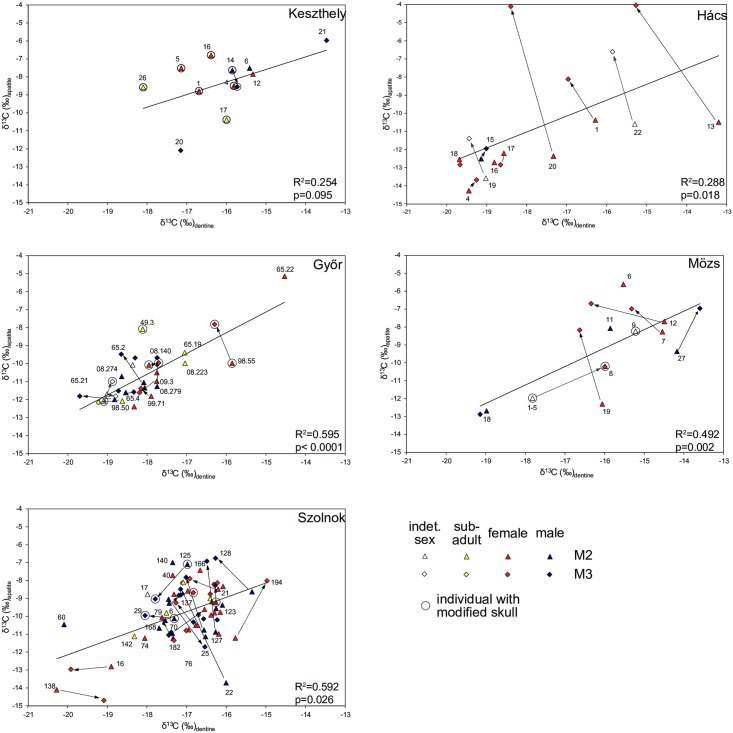
Scatter plots of δ^13^C_dentine_ and δ^13^C_apatite_ values at the five sites. Tooth pairs indicating lifetime changes in diet are marked with arrows. The trendlines visualise the correlation between δ^13^C_dentine_ and δ^13^C_apatite_, and the correlation coefficient R^2^ and p value for each are indicated. Data points are labelled with grave numbers.

Fig 5 shows δ^13^C dentine and enamel carbonate results, as well as tooth pairs. All sites exhibit a correlation between δ^13^C_dentine_ and δ^13^C_apatite_, though the relationship is not very strong. This is partly an artefact of small sample numbers. Where data points fall significantly above the trendline, indicating greater levels of δ^13^C_apatite_ enrichment relative to their δ^13^C_dentine_ values, we can assume that protein needs were met with animal protein and that δ^13^C_apatite_ enrichment was due to direct consumption of C4 plants.

This is most obviously the case in Hács, where the M3 of HAC20 and HAC13 were significantly enriched in δ^13^C_apatite_. These individuals, adult women, also exhibit a big difference between their M2 and M3. Their M2 were enriched in δ^13^C_dentine_, but not particularly elevated in δ^13^C_apatite_. This indicates that their dietary protein, possibly animals foddered on millet or C4 grasses, was a source of ^13^C enrichment. In Keszthely, in comparison, ^13^C contributions from protein and whole diet are fairly balanced. KFP5 and 26 are somewhat enriched in δ^13^C_apatite_, while KFP17 and 20 are more depleted, relative to δ^13^C_dentine_, but not very notably. Győr, similarly, has a tight correlation. Here, too, dietary contributions appear to be balanced. GYS65.22, a possibly female adult, stands out as being highly enriched in both δ^13^C_dentine_ and δ^13^C_apatite_. At Mözs the small group identified in Fig 4 (MOZ6, 7, 12, 27) also stands out. The M2 of the adult female MOZ6 is somewhat enriched in δ^13^C_apatite_. The M3 of the adult female MOZ12 is depleted in δ^13^C_dentine_, compared to her M2 but is slightly enriched in δ^13^C_apatite_, possibly reflecting residual C4 consumption. Szolnok, finally, also has a reasonably good correlation, though a few samples are outliers from the main distribution. SZO22, an adult male, was not obviously different in Fig 4, but stands out here because its M2 is enriched in δ^13^C_dentine_ and depleted in δ^13^C_apatite_. This may due to δ^13^C enriched animal protein consumption.

A comparison of δ^13^C contributions from protein and whole diet at the five sites also reveals significant lifetime shifts in some individuals. We can detect more subtle changes in different contributions to diet, but here too there are no obvious underlying demographic or archaeological patterns. The picture that emerges is one of societies with extremely heterogeneous dietary practices.

### Mobility

**Fig 6:**
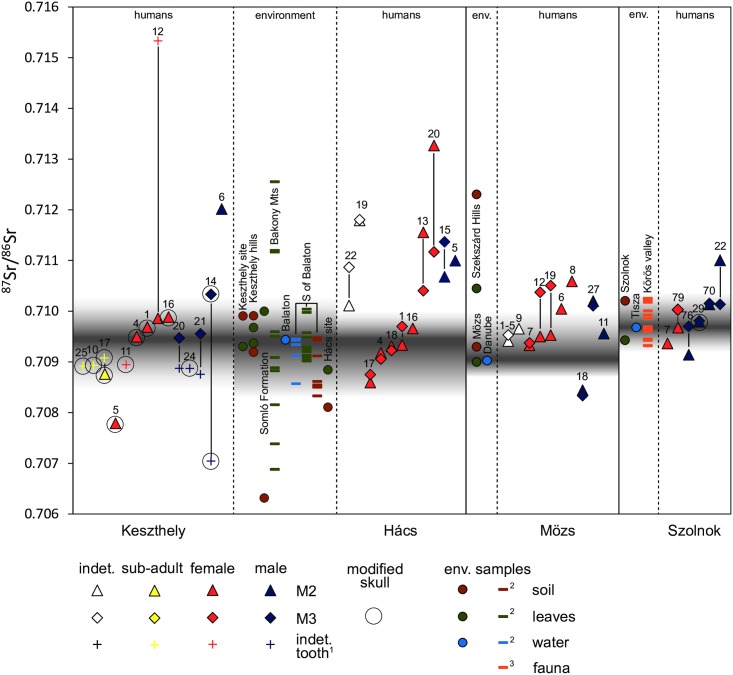
^87^Sr/^86^Sr ratios of human tooth enamel from Keszthely, Hács, Mözs and Szolnok represented in relation to modern vegetation, water, and soil samples and archaeological fauna. Tooth pairs are visualised with lines joining them. The shaded areas indicate local ^87^Sr/^86^Sr values. Shading extends from water samples (Balaton, Danube, Tisza), considered to be geological averages, to cover the local environmental variation. The environmental reference data for Keszthely and Hács are plotted between the human data of the two sites to show that they are relevant for both. For Mözs and Szolnok the reference data are shown to the left of the human data. Comparative data taken from published literature: ^1 87^Sr/^86^Sr ratios from an earlier study of some of the same individuals at Keszthely. The type of tooth used here could not be determined [92]; ^2^ comparative environmental data for the Balaton Region from [93]; ^3^ prehistoric fauna from the Körös valley, a tributary of the Tizsa [94].

Fig 6 shows the ^87^Sr/^86^Sr values of humans, plotted against the respective local environmental values. The shaded areas provide an estimate of the ^87^Sr/^86^Sr ratios in the vicinity of the sites. Water samples (Balaton, Danube and Tizsa) are taken to be local geological averages of ^87^Sr/^86^Sr, and from there the shading extends into the environmental variation at the sites. This variation is due to the choice of sampling location (intended to take account of geological variation) as well as to the way soils and plants interact with the local bedrock. S1 Fig shows the sampled locations and local geology in detail.

At Keszthely and Hács our own environmental samples are supplemented by the comprehensive soil, water and vegetation samples taken by Alt et al. [93] for the sixth-century cemetery of Szolad, south of Lake Balaton. The region around Lake Balaton is geologically highly variable. Fig 6 shows the ^87^Sr/^86^Sr values at the two sites, as well as at some distinct geological areas in the region (S1 Fig). Lake Balaton water can be assumed to have ^87^Sr/^86^Sr values that are a local average. The Bakony Mountains to the north of the lake are highly radiogenic, while the values around Keszthely and Hács (south of Lake Balaton) are lower. Human samples at Keszthely also include seven teeth previously analysed by Heinrich-Tamáska and Schweissing [92]. These teeth have not been identified.

At Mözs, the local ^87^Sr/^86^Sr ratios were defined by water from the Danube as well as by soil and vegetation samples. The more radiogenic data points represent the Szekszárd Hills. In Szolnok the local values are also determined by soil and vegetation from the site, and water from the Tisza, as well as by prehistoric fauna values from the Körös River, a tributary of the Tisza, published in Giblin [94]. Győr was excluded at this point to maximise available resources.

There is evidence of high levels of mobility at all four sites, both in terms of numbers of individuals with values falling outside the range defined as local to the site and in terms of the wide range of ^87^Sr/^86^Sr values. The data are not normally distributed and the sample size is small which necessitates visual analysis over statistical analysis. Some of the variability evident at Keszthely and Hács likely relates to the differences in ^87^Sr/^86^Sr ratios of the regional geology. The individuals with values outside of the range specific to the site may nevertheless have grown up in the Balaton region. There appears to have been considerable mobility within the region, if not from more distant areas. Only the values of the tooth from grave 12 in Keszthely and of the M2 from grave 20 in Hács fall completely outside of regional values, indicating that they did not grow up in the region. Several individuals are notable for extreme differences in the values of two teeth, suggesting that they lived at multiple locations during their childhood and youth.

At Mözs the local values represent the Danube floodplain and are therefore more closely constrained. Here, five of ten (graves 6, 8, 12, 19 and 27) sampled individuals fall outside of this range, but they could have resided locally in the more radiogenic area of the Szekszárd Hills before moving to Mözs at the end of their lives. The individual from grave 18 falls completely outside the regional ^87^Sr/^86^Sr values, indicating migration from some considerable distance. The underlying geology of Szolnok and surrounding areas, finally, is very homogeneous, being in the great loess basin of the Great Hungarian Plain. Here also two or perhaps three of the six analysed individuals exhibit non-local values (graves 22, 76 and possibly 7).

**Fig 7:**
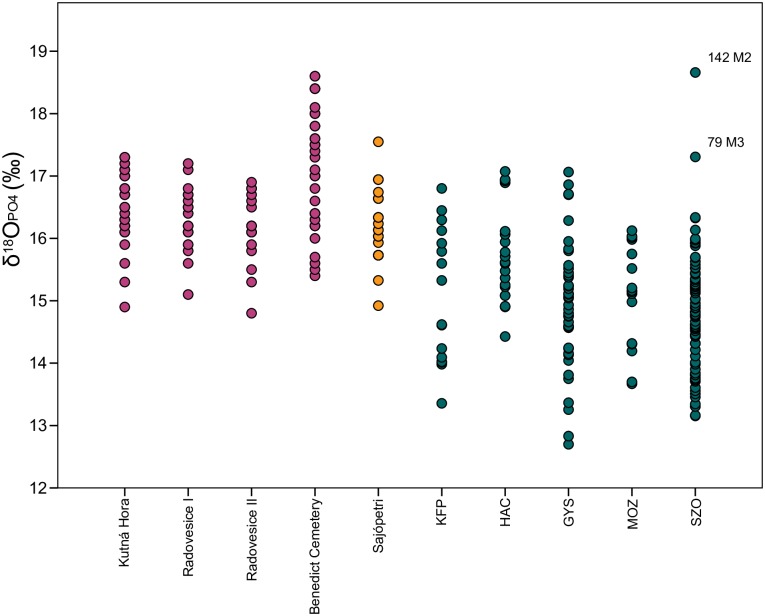
Comparison of δ^18^O values at different sites. The colours indicate the region where the sites are located. Pink: Bohemia; orange: northern Hungary; green: data from this study. Comparative data taken from published literature: Kutná Hora and Radovesice I and II (fourth to third century BC) [96]; St. Benedict Cemetery, Prague (fifteenth to eighteenth century AD) [98]; Sajópetri (Avar-period, seventh to ninth century AD) [95]; KFP—Keszthely-Fenékpuszta; HAC—Hács-Béndekpuszta; GYS—Győr; MOZ—Mözs; SZO—Szolnok-Szanda. Outliers are labelled with grave numbers. Columns are labelled as M2 –second molar and M3 –third molar.

Fig 7 shows the δ^18^O values from the five sites after conversion from their original carbonate values to phosphate values. They are compared against results from three Iron Age sites in Bohemia (Kutná Hora, Radovesice I and II), a post-medieval cemetery in Prague (St Benedict’s Cemetery) and an Avar-period cemetery in northern Hungary (Sajópetri [95–97]. The Bohemian populations and that of the cemetery in northern Hungary range from 14.9 to 17.8‰. A number of the individuals from the St Benedict Cemetery in Prague are more enriched, extending to 18.6‰, likely due to the greater mobility of this urban population, and because they may have had greater access to cooked and stewed foods [42]. The five sites of our study are more depleted in δ^18^O, possibly reflecting local water sources. While the distributions appear uneven, the data are normally distributed, and only Szolnok includes two clear outliers (79 and 142). These can be considered non-local to Szolnok with some degree of certainty. The data ranges are greater than those at the three Iron Age sites, and more comparable to the St Benedict Cemetery. This supports the evidence for high levels of mobility already indicated by the ^87^Sr/^86^Sr values.

The ^87^Sr/^86^Sr and δ^18^O values indicate high levels of mobility at all sites. While the distances travelled since childhood cannot be determined, it is clear that the cemetery populations had heterogeneous biographies. Some individuals experienced more than one change in residence. There are no clear patterns here, so it is unlikely that they resided in the same locations and travelled as groups. Just as with the dietary patterns discussed above, levels of mobility are independent from sex, the presence of skull modification or specific elements of the burial practice. As expected, younger individuals more commonly, though not always, have local signatures.

## Discussion

At all five sites humans consumed a medium to high amount of animal protein, higher than at the comparison sites in Germany, but lower than at most of the Inner Asian sites. The protein appears to have derived from terrestrial, rather than aquatic, fauna. The fish sampled here are mostly enriched in ^15^N compared to terrestrial fauna, though carps and cyprinids have similar δ^15^N values (S3 Fig). With the exception of the catfish from Győr and Esztergom, fish are depleted in ^13^C compared to other fauna. Since fish are higher in protein than millet, we should expect them to affect the human values more strongly. Any significant contribution would therefore lead to lower δ^13^C values, though some of this would be cancelled out by millet consumption.

The five Hungarian sites fall between the German and Inner Asian sites in their δ^13^C values. This indicates significant, though not exclusive, consumption of C4 plants, most likely millet. Millet has been recorded archaeobotanically in late Roman Pannonia, where it was relied upon as a minor crop among a wider range of cereals [78, 99]. Significantly, archaeobotanical analysis at Keszthely of samples from multiple contexts within the fortification revealed multi-rowed barley to be the main crop, followed by oats, broomcorn millet, rye and wheat [79]. This contrasts with numerous third- to fifth-century sites beyond the frontier where significant amounts of millet were found, often combined with barley [85, 99]. It is possible that millet increased in the fourth and fifth centuries due to population movements from and contacts with Central Asia where this grain was more widespread.

At the four sites where strontium isotope analysis was carried out, between thirty and fifty percent of sampled individuals were not local to the area where they were buried. The populations were extremely heterogeneous. Places of origin are variable and, in many cases, individuals underwent more than one change in residence. Additionally, it is not possible to identify groups that may have moved together. Remarkably, all five sites broadly exhibit the same patterns, regardless of their location within or beyond the late Roman frontier. Szolnok is in the Great Hungarian Plain, the most likely region to support a pastoral lifestyle, yet it exhibits similar patterns to the other four sites.

A detailed examination of lifetime changes shows that this variability has a high resolution. It is difficult to ascertain what degree of change within the same individual can be attributed to ‘normal’ dietary variation. Incremental dentine studies have revealed changes of Δ^15^N 3 to 5‰ in a single human tooth [46, 47]. Even bulk dentine samples record diet over a shorter time-period than bone collagen, making dentine samples more sensitive to short-term dietary changes. While elevated δ ^15^N can represent both nutritional stress, a metabolic relationship with δ^13^C is limited. We can thus assume that variations in δ^13^C values are a reflection of differential contributions of C3 and C4 plants. Many individuals therefore experienced dietary changes and changes in residence over only a few years, though lifetime variability in diet is not directly linked to high levels of mobility in all cases.

Taken together, the evidence suggests that our sampled populations followed subsistence practices that were neither exclusively agricultural nor fully pastoral. Most of their protein derived from domesticates such as cattle, ovicaprids and possibly also horses and pigs. They engaged in agriculture to some degree, either directly or through trade and exchange with agricultural populations, and much of grain production focused on millet. Levels of mobility are consistent with some degree of nomadism, and many individuals appear to have switched subsistence strategies over the course of their lives.

## Conclusion

The isotopic evidence reveals a picture of great dietary variability, both within populations and over individuals’ lifetimes. The patterns at all sites suggest subsistence strategies that include medium to high animal protein consumption with little evidence for a significant contribution of aquatic resources. All populations relied to great extent on C4 plants, most likely millet. Within each population, diet was heterogeneous, with significant variations in terms of animal protein and C3 and C4 plant consumption. High levels of intra-population and individual variability suggest that populations made use of a range of subsistence strategies, with many individuals exhibiting significant changes over their lifetimes.

It is therefore likely that groups and individuals switched between subsistence strategies, often quite rapidly. This may have been a response to a period of warfare and instability in the frontier zones, as recorded in historical sources. Certainly agricultural diversification is a safer strategy than reliance on monoculture in uncertain economic times. Conversely, switching of subsistence strategies may have been a proactive choice. Most pastoralists rely on some form of agriculture, either by engaging in it themselves or through close interaction with agricultural populations, and nomadic groups can be mobile for part of a year or move between long-established locations. Such a fluid approach to apparently very different subsistence strategies has been documented ethnographically (e.g. [100, 101, 102]), but also archaeologically [14].

The burial evidence—grave construction and grave goods—from the four cemeteries within Pannonia is largely in keeping with wider developments in middle and lower Danubian burial practice in the fifth century [63, 103]. There is no evidence for great social disruption or economic deprivation. Graves were commonly well-constructed and fairly well-furnished. The cemetery at Keszthely was associated with the late Roman fortification, but self-contained and at some distance from it [57]. In Győr the graves were located within the former Roman vicus following its abandonment [67]. Hács-Béndekpuszta and Mözs, on the other hand, were newly established, though a number of graves at Mözs were brick-lined, in the late Roman tradition. Similarly, the burial evidence at Szolnok-Szanda is in keeping with local practices [73]. While some grave goods display long-distance connections to the Pontic regions (e.g. shoe buckles in Mözs, grave 11), but also to west-central Europe (e.g. the brooches in Keszthely, grave 10, and Hács, graves 19 and 20 with a parallel in south-west Germany), the funerary context does not suggest that particular individuals were considered foreigners or outsiders. Nor are individuals with outlying isotopic data marked out as different in their burials. The funerary evidence thus suggests that the individuals or groups that newly arrived in the areas where they were buried were largely part of the hybridised environment of late antique Pannonia and the plains beyond the frontier rather than outsiders that arrived from regions as far as central Asia, as has been suggested [104]. Instead, it is likely that the steppe environments of the Carpathian basin and the Pontic littoral generated the phenomenon of the Huns locally. The archaeological and isotopic evidence presented here therefore does not identify ‘Huns’ as a distinct ethnic group. Instead it reveals the impact of nomadic-pastoralist groups on the populations of the Carpathian basin.

The environment of the Carpathian basin was a mosaic of steppe, open forests and agricultural land. This makes it likely that the inhabitants would have relied on both agriculture and pastoralism to suit their needs. Interestingly this was the case at the sites within late Roman Pannonia, as well as in Szolnok, in the Great Hungarian Plain, the ecological region most likely to support large-scale animal herding. At Keszthely, Hács and Győr, we may have evidence of fairly transient populations. The cemetery at Mözs is much larger than the sub-sample included in this study and has a large associated settlement. Nevertheless, it is possible that a number of those buried there had not settled there long-term. At Szolnok, we have no associated settlement. Chronologically it spans the fifth and sixth centuries, a longer period than the other sites. Its size and period of use suggests a fairly large, relatively stable population. Here it may be possible that some individuals engaged in pastoralism within a mostly sedentary population.

While written accounts of the last century of the Roman empire may document particular convulsions of violence, they are largely silent on the cooperation and coexistence of people living in the frontier zone. Our new isotopic evidence shows that populations along the late Roman frontier in Hungary adopted a highly flexible approach to subsistence, in keeping with high levels of hybridity evident in burial practice. Farming and animal herding were not fundamentally opposed to each other, but mutually beneficial strategies that were not limited to particular ethnic groups. Nomadic-pastoralist groups may have switched to smaller herds and more farming, and, conversely, local populations may have integrated with a new economic system based on animal herding, and these changes could happen over the course of a person’s life. The influx of nomadic populations into east-central Europe in the fifth century AD may have caused enormous political upheaval and documented episodes of violence, but isotopic evidence shows people finding strategies to mitigate and perhaps even to benefit from these changes by modifying their subsistence economies.

## Supporting information

S1 Text Analytical methods. (PDF)

S2 Text Sampling strategy. (PDF)

S1 Table Tooth formation patterns in humans. (PDF)

S2 Table Full isotopic results. (XLSX)

S3 Table Summary isotopic data for cattle and ovicaprids at the five sites. (PDF)

S1 Fig Geological environment of (A) Keszthely-Fenékpuszta and Hács-Béndekpuszta; (B) Mözs and (C) Szolnok-Szanda, showing locations were reference samples for ^87^Sr/^86^Sr were taken. (TIF)

S2 Fig Scatterplots of isotopic results of δ^13^C and δ ^15^N for cattle and ovicaprids at each site. The data points with error bars represent the mean with one standard deviation.
(TIF)

S3 Fig Scatterplots of isotopic results of δ^13^C and δ ^15^N of humans and fauna at each site. (TIF)
