# Supplementary material for: Practising pastoralism in an agricultural environment: An isotopic analysis of the impact of the Hunnic incursions on Pannonian populations
Source: PLoS One. 2017 Mar 22;12(3):e0173079. doi: 10.1371/journal.pone.0173079 (PMC5362200; doi:10.1371/journal.pone.0173079)
Supplement: S1 Text — (PDF) [file pone.0173079.s001.pdf]

S1 Text: Analytical methods

Carbon and nitrogen isotope analysis of collagen and dentine

Tooth roots were cut at the crown, resulting in dentine samples of 0.2-0.4 g. Human bone samples (0.5-1.5 g) were taken from ribs where available, otherwise from long bones. Samples from fauna were more variable, though cortical bone was preferred. One of the fish samples (KFP F026) was made up of small fish bones taken in bulk.

Collagen was extracted following the standard laboratory protocol of the Department of Archaeology and Anthropology, University of Cambridge, based on the method detailed by Privat et al. [1]. The surfaces of the bone pieces were cleaned by sand blasting. The pieces were demineralized in 0.5 M aq. HCl at 4°C until all mineral had dissolved (7-14 days). Samples were rinsed with distilled water and then gelatinized in acidic solution (pH 3) at 75–78°C for 48h. The liquid fraction containing the gelatinized protein was frozen and lyophilised to produce the final 'collagen' product. Approx 0.8mg portions of this 'collagen' were used for each analysis. Samples were analysed in triplicate at the Godwin Laboratory, University of Cambridge, using an automated elemental analyser (Costech) coupled in continuous flow mode to an isotope ratio-monitoring mass spectrometer (Finnegan MAT253). Results are reported using the delta scale in units of 'per mil' (‰) relative to internationally accepted standards, VPDB for carbon and AIR for nitrogen. Measurement errors are less than ±0.2‰ for $\delta^{13}C$ and $\delta^{15}N$, as determined by replicate analyses of international and laboratory standards.

Carbon and oxygen isotope analysis of enamel bioapatite

Tooth enamel powder was obtained using a using a dental drill with a diamond drill attachment. The exterior of the enamel was mechanically abraded to remove any dirt, and the drill bit was cleaned before each sample was taken.

The bioapatite extraction method is described in Balasse et al. [2]. Enamel was treated with sodium hypochlorite 2–3% (24 h) to remove organic matter and then with 0.1 M acetic acid (4 h, 0.1 ml/mg) to remove exogenous carbonate. The samples were lyophilised to remove any remaining liquid. The samples were then transferred into vials sealed with a screw cap holding a septa and PCTFE washer to make a vacuum seal, and the samples reacted with 100% orthophosphoric acid at 90°C using a Micromass Multicarb Sample Preparation System. The carbon dioxide produced was dried and transferred cryogenically into a VG SIRA mass spectrometer for isotopic analysis. Results are reported with reference to the international standard VPDB calibrated through the NBS19 standard [3: 60, 4]. The precision is better than ±0.08‰ for $^{13}C/^{12}C$ and better than ±0.10‰ for $^{18}O/^{16}O$.

Strontium isotope analysis

Environmental samples for strontium analysis were pre-treated following the methods described in Maurer et al. [5]. Water samples were filtered through 0.2um nylon filters into 60ml acid cleaned HDPE bottles and acidified with distilled ultra-pure nitric acid to maintain a pH = 2.

Soil samples were taken with an auger to below the topsoil, reaching depths of 0.25 to 0.70 m. Fields under cultivation were avoided to mitigate the impact of fertiliser use. Soil leachates

were obtained by shaking 2 g of soil in 20 ml deionised ultrapure water for 24 h in acid-cleaned polypropylene tubes, followed by 1 h in an ultrasonic bath. The solution was centrifuged for 15 minutes at 2000 rpm and filtered through 0.2um nylon filters.

Water and soil leachates were evaporated to dryness. Leaves were rinsed with deionised ultrapure water and dried in an oven at 50C overnight. Approximately 1 g of dried leaves was then ground and ashed in acid-washed silica crucibles at 550C for 12 h. All samples were then transferred into acid-cleaned Teflon vials.

The enamel surface of the tooth was abraded to a depth of >100 microns using a tungsten carbide dental burr and the removed material discarded. Tooth enamel powder was then collected, and the samples were transferred to a clean (class 100, laminar flow) working area for further preparation. In a clean laboratory, the samples were first washed in high purity acetone to remove any grease that might have come from handling the enamel. Then the sample was cleaned ultrasonically in high purity water, rinsed, dried and then weighed into pre-cleaned Teflon vials. The samples were mixed with ^{84}Sr spike solution and dissolved in Teflon distilled 8M HNO3.

Environmental samples were analysed at the Isotope Geochemistry Laboratory of the Department of Earth Sciences, University of Cambridge; enamel was analysed at the NERC Isotope Geoscience Facilities, Keyworth.

For samples analysed at NIGL, Strontium was collected using Dowex resin columns. Strontium was loaded onto a single Re Filament with TaF following the method of [6], and the isotope composition and concentrations were determined by Thermal Ionisation Mass spectroscopy (TIMS) using a Thermo Triton multi-collector mass spectrometer. The international standard for 87Sr/86Sr, NBS987, gave a value of 0.710251 ± .000005 (n=19, 2 sigma) during the analysis of these samples. All analyses run to internal precision of better than ± 0.000014 (2SE). Blank values were in the region of 100pg.

Samples analysed in Cambridge for strontium were separated using Dowex 50 x 8 (200-400 mesh) cation exchange resin and the strontium isotopic ratios were measured on single Ta filaments on the VG Sector 54 TIMs using triple collector dynamic algorithm, normalised to $^{86}Sr/^{88}Sr$ 0.1194 with an exponential fractionation correction [7]. The 46 analyses of NBS 987 during the two year period up to and including these analyses gave a mean value of 0.710257+/-0.000006%(1sigma).

References

1. Privat KL, O'Connell TC, Richards MP. Stable Isotope Analysis of Human and Faunal Remains from the Anglo-Saxon Cemetery at Berinsfield, Oxfordshire: Dietary and Social Implications. Journal of Archaeological Science. 2002;29(7):779-90. doi: DOI: 10.1006/jasc.2001.0785.
2. Balasse M, Ambrose SH, Smith AB, Price TD. The seasonal mobility model for prehistoric herders in the south-western cape of South Africa assessed by isotopic analysis of sheep tooth enamel. Journal of Archaeological Science. 2002;29(9):917-32. doi: 10.1006/jasc.2001.0787.
3. Hoefs J. Stable Isotope Geochemistry. 6th ed. Berlin and Heidelberg: Springer-Verlag; 2009.
4. Coplen TB. New IUPAC guidelines for the reporting of stable hydrogen, carbon and oxygen isotope-ratio data. Journal of Research of the National Institute of Standards and Technology. 1995;100:285-.

5. Maurer A-F, Galer SJG, Knipper C, Beierlein L, Nunn EV, Peters D, et al. Bioavailable 87Sr/86Sr in different environmental samples — Effects of anthropogenic contamination and implications for isoscapes in past migration studies. Science of The Total Environment. 2012;433(0):216-29. doi: http://dx.doi.org/10.1016/j.scitotenv.2012.06.046.
6. Birck JL. Precision K-Rb-Sr Isotopic Analysis - Application to Rb-Sr Chronology. Chemical Geology. 1986;56(1):73-83. doi: http://dx.doi.org/10.1016/0009-2541(86)90111-7.
7. Bickle MJ, Bunbury J, Chapman HJ, Harris NBW, Fairchild IJ, Ahmad T. Fluxes of Sr into the headwaters of the Ganges. Geochimica et Cosmochimica Acta. 2003;67(14):2567-84. doi: http://dx.doi.org/10.1016/S0016-7037(03)00029-2.
