# Supplementary material for: Practising pastoralism in an agricultural environment: An isotopic analysis of the impact of the Hunnic incursions on Pannonian populations
Source: PLoS One. 2017 Mar 22;12(3):e0173079. doi: 10.1371/journal.pone.0173079 (PMC5362200; doi:10.1371/journal.pone.0173079)
Supplement: S2 Text — (PDF) [file pone.0173079.s002.pdf]

S2 Text: Samples and Sampling strategy

Following Maurer et al. [1], bioavailable strontium ratios were established by sampling water from rivers and streams, vegetation (tree leaves from areas not immediately near agriculture) and soil. To determine 'local' isotopic signatures of $^{87}Sr/^{86}Sr$, samples were collected at multiple locations around the south-west end of Lake Balaton and around Mözs to characterise the high degree of localised geological variability (Fig. S1). This complements work by Alt et al. [2] on the Lombard-period cemetery of Szólad, south of Lake Balaton. Other sites necessitated fewer sampling locations, due to the greater homogeneity of the local geology. Information about sample types and locations is provided in the supporting information (S2 Table).

Faunal remains are curated in the Hungarian Agricultural Museum, Budapest (1146 Budapest, Városliget, Vajdahunyadvár) where they can be accessed by researchers. Since the material is retained in bulk, no individual specimen numbers were assigned by the museum. S2 Table provides the site context numbers. Permission for destructive sampling was granted by the museum (MMGM/579/2013). Bones were selected from each site to provide a local environmental baseline. Due to the geographical proximity of Keszthely-Fenékpuszta and Hács-Béndekpuszta, the fauna from Keszthely was used to provide a baseline for both sites. At Győr-Széchenyi Square, fauna was primarily taken from graves where bones had either been found in the fill or had been deposited as a food offering and from the late Roman settlement features. For Mözs the fauna came from the associated settlement. At Szolnok no directly associated fauna could be located; instead samples were taken from a tenth-century cemetery [3]. Fish bones were accessed from the late Roman contexts at Keszthely-Fenékpuszta, as well as from the early Neolithic site at Szajol, near Szolnok [4], the late Chalcolithic site of Gyöngyöshalász-Encspuszta [5] and Árpád-period (tenth to thirteenth century AD) Esztergom on the Danube bend [6], for general reference. At all sites fauna was selected broadly to represent the range of species found there.

Human remains are housed in the anthropological collection of the Hungarian Natural History Museum Budapest (1083 Budapest, Ludovika tér 2-6) where they can be accessed by researchers (inventory numbers: Keszthely-Fenékpuszta 2009.26.1-31; Hács-Béndekpuszta: 8877-8880, 9314-9321, 9439-9441; Mözs: 11.874-896, 12815; Szolnok-Szanda 7768-7868; 68.57.1-15; Győr-Széchenyi Square: 1949.3, 65.1-23, 1998.50-1999.89, 2008.1-2009.436). Permission for destructive sampling was granted by the museum (dated 16/05/2012). The grave numbers used in the present study (S2 Table) are also used by the museum. Samples were taken from all individuals where the relevant teeth and rib bones were available. For carbon and nitrogen analyses the aim was a 100% sample. For strontium analysis, samples were limited to individuals where multiple tissues were available. Győr was excluded at this stage. Information about the age and sex of humans was taken from the associated publications. For Győr (not yet fully published) this was carried out according to the methods described in [7, 8].

1. Maurer A-F, Galer SJG, Knipper C, Beierlein L, Nunn EV, Peters D, et al. Bioavailable 87Sr/86Sr in different environmental samples — Effects of anthropogenic contamination and implications for isoscapes in past migration studies. Science of The Total Environment. 2012;433(0):216-29. doi: http://dx.doi.org/10.1016/j.scitotenv.2012.06.046.
2. Alt KW, Knipper C, Peters D, Müller W, Maurer A-F, Kollig I, et al. Lombards on the Move – An Integrative Study of the Migration Period Cemetery at Szólád, Hungary. PLoS ONE. 2014;9(11). doi: 10.1371/journal.pone.0110793.

3. Madaras L. Szolnok, Lenin TSZ (ugar) 10. századi temtöje (Der Begräbnisplatz aus der Zeit der Landnahme in der Zentrale der Szolnoker "Lenin" LPG). In: Wolf M, Révész L, editors. A Magyar honfoglalás korának régészeti emlékei. Miskolc1996. p. 65-116.
4. Vörös I. Zoological and palaeoeconomical investigations on the archaeozoological material of the early Neolithic Körös culture. Folia Archaeologica. 1980;31:35-64.
5. Takács I. Gyöngyöshalász-Encspuszta késő rézkori település halcsont leleteinek elemzése. Agria. 1982-1983;19:63-73.
6. Vörös I. Esztergom-Szentgyörgymező Árpád-kori település állatcsontmaradványai Előzetes közlemény (Tierknochenfunde aus der árpádenzeitlichen Siedlung von Esztergom-Szentgyörgymező. Vorbericht). Dunai Régészeti Kozlemények. 1989;3:51-6.
7. Brothwell DR. Digging up bones: the excavation, treatment, and study of human skeletal remains. 3rd ed., rev. and updated. ed. Ithaca, N.Y.: Cornell University Press; 1981.
8. Schinz H, Baensch W, Friedl E, Uehlinger E. Ossifikationstabelle. In: Schinz HR, editor. Lehrbuch der Röntgendiagnostik. 5 ed. Stuttgart: Thieme; 1952. p. 1-4.
