# Supplementary material for: Practising pastoralism in an agricultural environment: An isotopic analysis of the impact of the Hunnic incursions on Pannonian populations
Source: PLoS One. 2017 Mar 22;12(3):e0173079. doi: 10.1371/journal.pone.0173079 (PMC5362200; doi:10.1371/journal.pone.0173079)
Supplement: S1 Table — (PDF) [file pone.0173079.s003.pdf]

S1 Table. Tooth formation patterns in humans

	Tooth	Crown begins to form	Crown complete	Root complete
**Maxilla**	**P2**	2-2.25	6-7	12-14
	**M2**	2.5-3	7-8	14-16
	**M3**	7-9	12-16	18-25
**Mandible**	**P2**	2.25-2.5	6-7	13-14
	**M2**	2.5-3	7-8	14-15
	**M3**	8-10	12-16	18-25

Ages given in years
