# Supplementary material for: Practising pastoralism in an agricultural environment: An isotopic analysis of the impact of the Hunnic incursions on Pannonian populations
Source: PLoS One. 2017 Mar 22;12(3):e0173079. doi: 10.1371/journal.pone.0173079 (PMC5362200; doi:10.1371/journal.pone.0173079)
Supplement: S3 Table — (PDF) [file pone.0173079.s005.pdf]

S3 Table. Summary isotopic data for cattle and ovicaprids at the five sites

	δ^{13}C (‰)					δ^{15}N (‰)					
	**mean**	**1σ**	**min**	**max**	**range**	**mean**	**1σ**	**min**	**max**	**range**	**n**
**Keszthely:**											
**fauna:**											
**Bos**	-20.1	0.7	-21.0	-18.8	2.3	6.3	0.8	5.1	7.3	2.3	5
**Ovicaprid**	-20.4	0.4	-20.6	-19.7	1.0	7.1	1.1	6.3	9.0	2.7	4
**combined**	-20.2	0.6	-21.0	-18.8	2.3	6.7	1.0	5.1	9.0	3.9	9
**Győr:**											
**fauna:**											
**Bos**	-19.0	1.5	-20.9	-16.7	4.2	7.1	0.8	5.3	8.0	2.7	8
**Ovicaprid**	-20.1	1.2	-21.4	-18.0	3.4	8.0	1.7	5.7	10.8	5.1	6
**combined**	-19.4	1.5	-21.4	-16.7	4.7	7.5	1.4	5.3	10.8	5.5	14
**Mözs:**											
**fauna:**											
**Bos**	-19.8	0.9	-20.8	-17.7	3.1	6.9	0.6	5.8	7.9	2.0	7
**Ovicaprid**	-20.5	0.2	-20.8	-20.4	0.4	7.5	0.4	7.0	8.0	1.0	4
**combined**	-20.1	0.8	-20.8	-17.7	3.1	7.1	0.6	5.8	8.0	2.2	11
**Szolnok:**											
**fauna:**											
**Bos**	-20.2	0.4	-20.9	-19.7	1.2	7.6	0.4	7.1	8.2	1.0	5
**Ovicaprid**	-20.2	0.6	-21.1	-19.0	2.1	8.2	0.8	6.9	9.1	2.2	7
**combined**	-20.2	0.6	-21.1	-19.0	2.1	7.9	0.8	6.9	9.1	2.2	12
