# Supplementary figures and images for: Practising pastoralism in an agricultural environment: An isotopic analysis of the impact of the Hunnic incursions on Pannonian populations
Source: PLoS One. 2017 Mar 22;12(3):e0173079. doi: 10.1371/journal.pone.0173079 (PMC5362200; doi:10.1371/journal.pone.0173079)

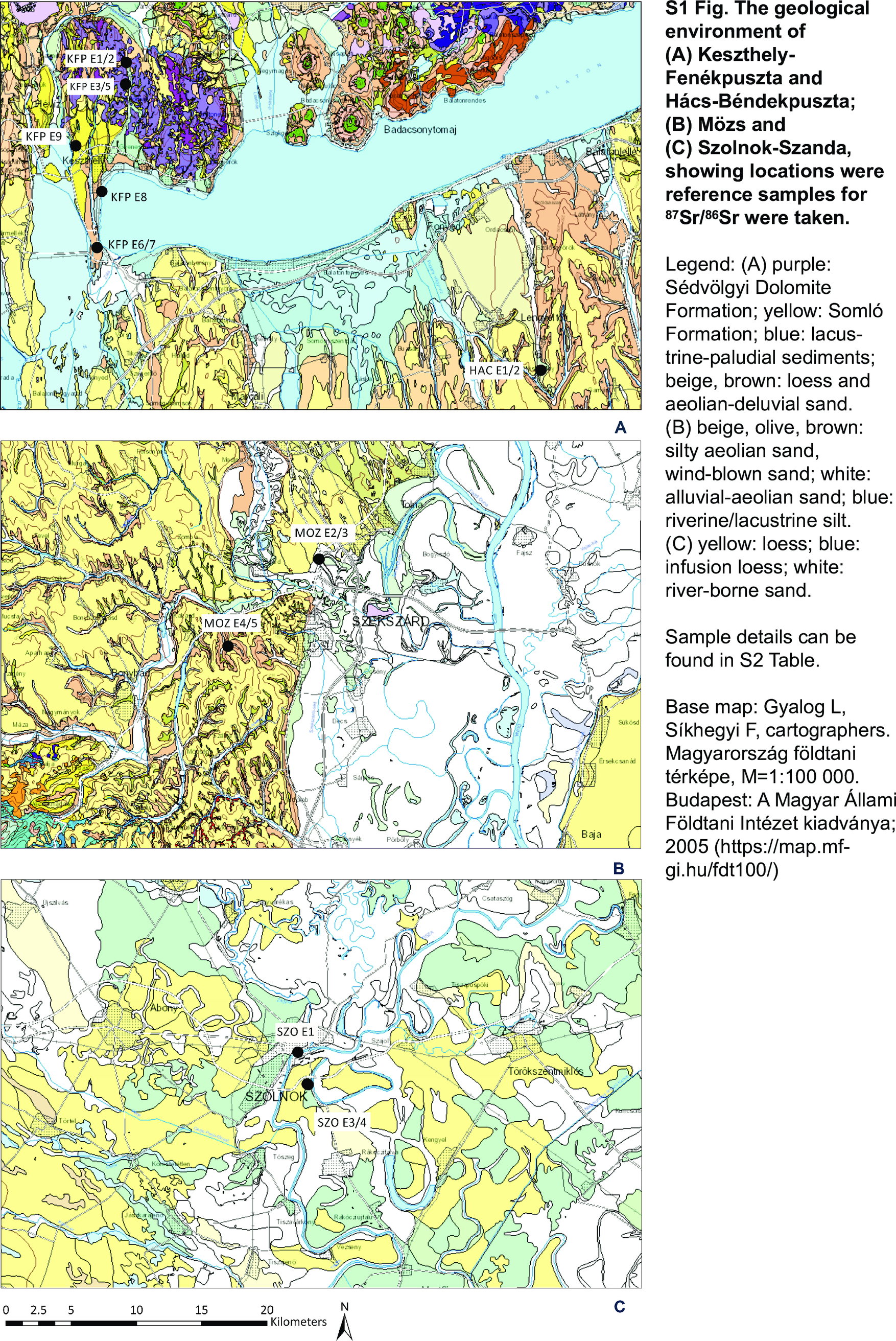

Supplement: S1 Fig — (TIF) [file pone.0173079.s006.tif]

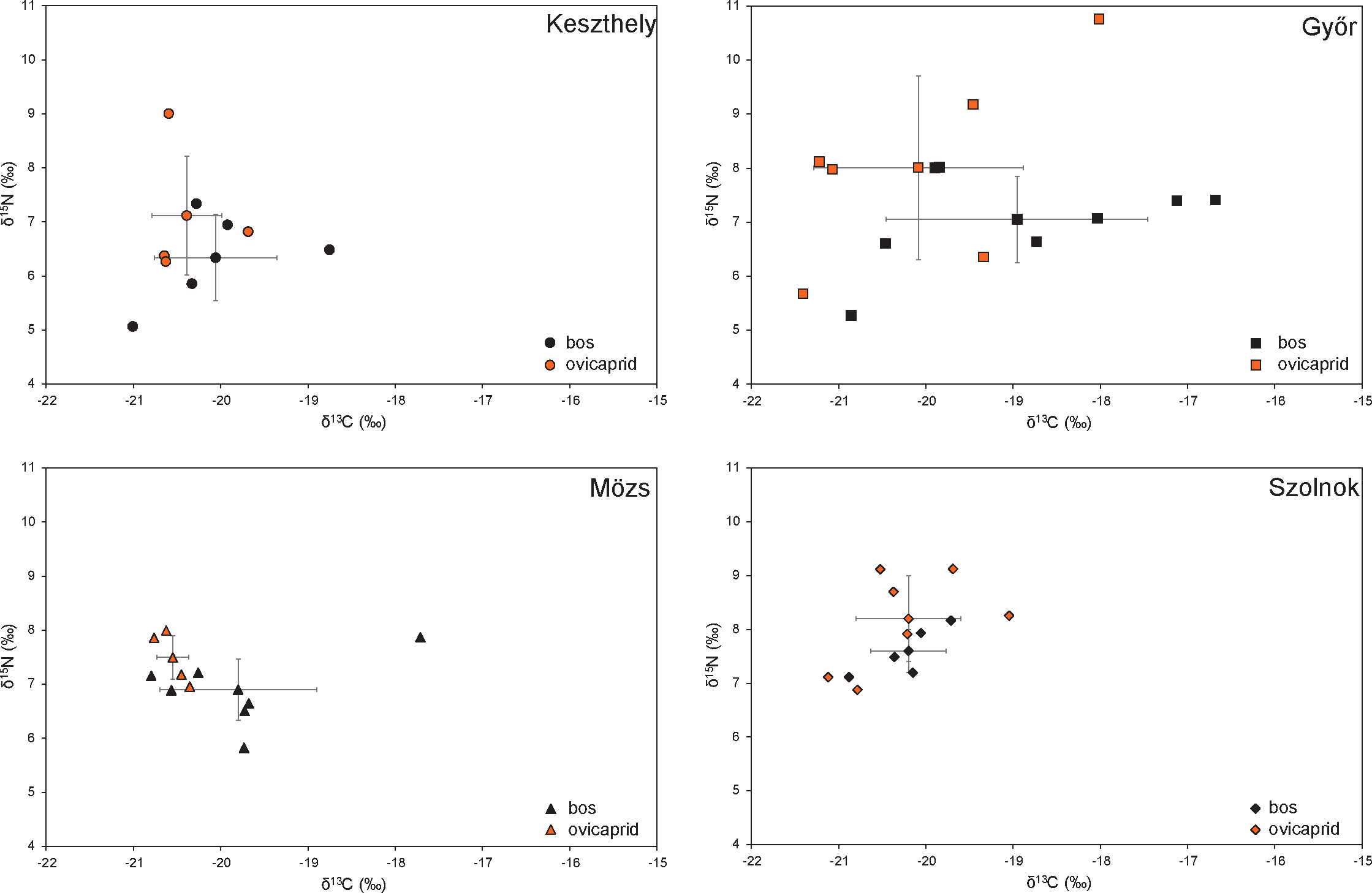

Supplement: S2 Fig — The data points with error bars represent the mean with one standard deviation. (TIF) [file pone.0173079.s007.tif]

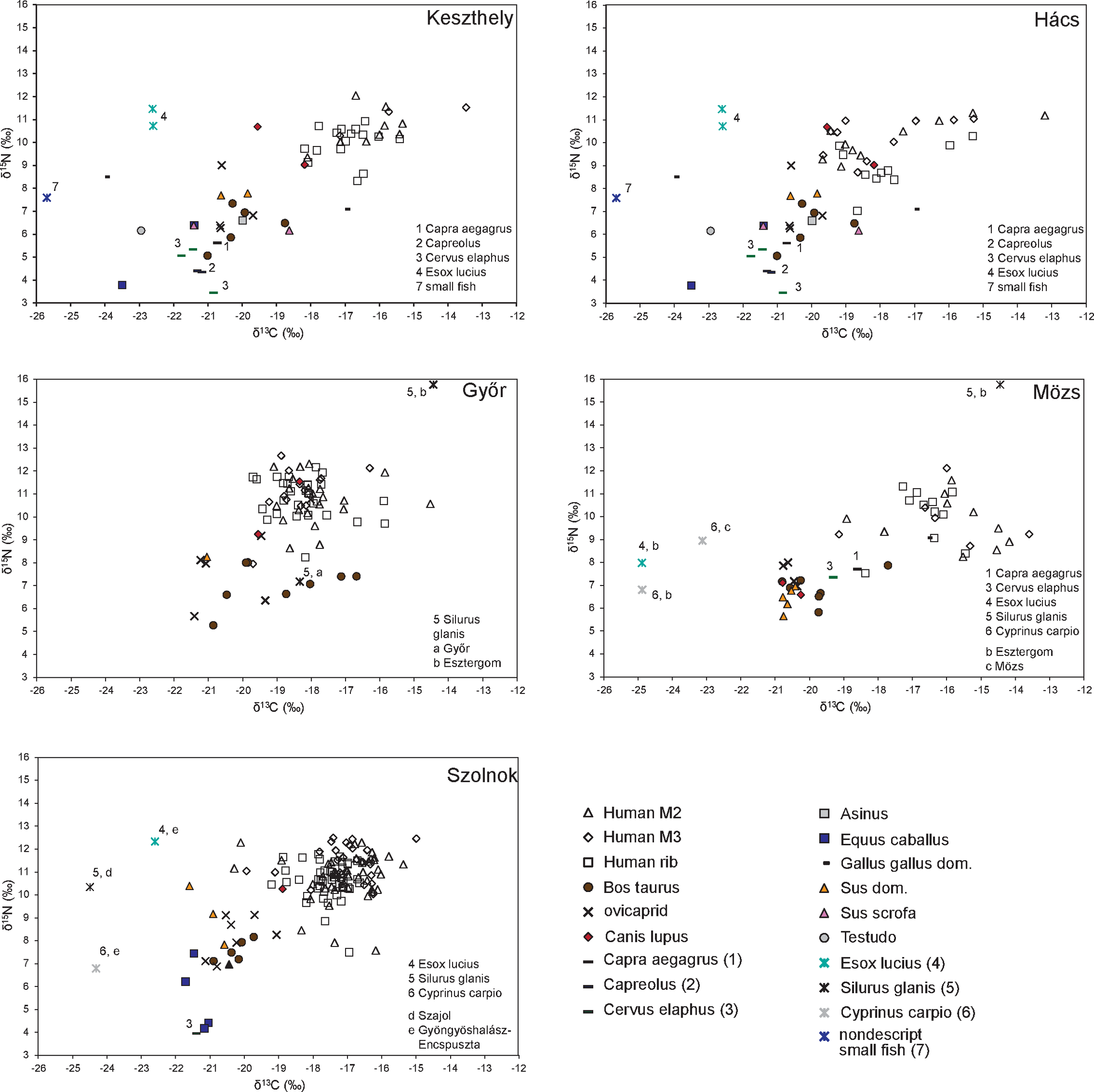

Supplement: S3 Fig — (TIF) [file pone.0173079.s008.tif]
